# Recent advances in therapies for eating disorders

**DOI:** 10.12688/f1000research.19847.1

**Published:** 2019-09-26

**Authors:** Lauren E. Davis, Evelyn Attia

**Affiliations:** 1 New York State Psychiatric Institute, Columbia University Irving Medical Center, New York, USA; 2 Weill Cornell Medical Center, New York, USA

**Keywords:** Eating disorders

## Abstract

Eating disorders are serious psychiatric illnesses with high rates of morbidity and mortality. Effective treatments have traditionally included behaviorally focused therapies as well as several medication strategies. Recent years have seen promising developments in these treatments, including additional support for family-based approaches for children and adolescents, new evidence for “third-wave” behavioral therapies, and new support for the use of lisdexamfetamine for binge eating disorder and olanzapine for anorexia nervosa. Case study and pilot data are beginning to show limited support for neuromodulatory interventions targeting brain regions thought to be involved in eating disorders. This review summarizes treatment developments over the last several years and points towards future directions for the field.

## Introduction

Eating disorders, including anorexia nervosa (AN), bulimia nervosa (BN), binge eating disorder (BED), and avoidant-restrictive food intake disorder (ARFID), are serious psychiatric illnesses associated with high rates of morbidity and mortality. Recent epidemiological data from a United States population estimated lifetime prevalence rates of 0.80% (AN), 0.28% (BN), and 0.85% (BED), though estimates vary across studies ^1, 2^. Treatments are frequently multi-faceted, with psychotherapy and pharmacotherapy being commonly utilized across a range of treatment settings. Recent treatment advances in psychotherapies were previously highlighted in a review ^3^, focusing on the advancement of cognitive-behavioral treatments for BN and BED as well as family-based therapy (FBT) for children and adolescents with AN. Since then, behavioral therapies have continued to flourish and expand, integrating the transdiagnostic model of cognitive-behavioral therapy (CBT) into clinical practice. While BN has the most empirical support for pharmacologic interventions, recent studies have shown support for medications involved in the treatment of BED and AN, including one FDA-approved drug to treat BED.

Across the spectrum of eating disorders, there is still much to be learned about their etiology, their development, and ways in which they can be effectively managed and treated. Recent developments in neuroscience and neuroimaging have begun to identify possible treatment targets for eating and other psychiatric disorders. The neurocircuitry that may be responsible for disturbances in eating behavior has provided the field with new ideas about contributing factors to the development and maintenance of eating disorders and possible treatments for these disturbances.

This review aims to summarize findings from recent studies of psychological, medicinal, and other adjunctive treatments that have been used for individuals with eating disorders. Randomized controlled trials (RCTs) as well as more preliminary reports including open series will be described.

## Psychotherapies

The transdiagnostic approach to understanding core cognitive and behavioral features co-occurring across eating disorder diagnoses continues to shape the various psychotherapies in practice today. In recent years, Fairburn and colleagues built upon their initial work developing and studying CBT for BN and BED, creating a transdiagnostic version of CBT (CBT-E) ^4^. This manualized treatment uses many of the familiar principles of CBT (e.g. food monitoring, cognitive challenges, etc.) but includes treatment components that specifically target different eating behaviors in order to have relevance across the range of different eating disorders.

A 2015 study ^5^ randomized 130 transdiagnostic patients (including patients with BN, BED, and “other eating disorder”) to either CBT-E or interpersonal psychotherapy (IPT). Levels of general and eating disorder-specific psychopathology were assessed post-treatment, along with BMI and presence of binge eating/purging behaviors. Overall, levels of psychopathology decreased across both groups but with a significantly greater change among those randomized to CBT-E. A greater proportion of patients who received CBT-E achieved remission while in treatment (as defined by a global eating disorder examination score below 1.74) and a greater percentage of patients in CBT-E versus IPT (44.8% versus 21.7%) reported no binge eating, vomiting, or laxative misuse post-treatment. These findings strengthen the evidence base for using cognitive-behavioral treatments for BN and BED and begin to support its use in higher-weight patients with binge eating and purging behaviors.

Two recent meta-analyses ^6, 7^ have further supported the use of CBT for the treatment of both BN and BED. Hilbert and colleagues found that for BED, RCTs comparing CBT to inactive control groups found a significantly reduced number of binge eating episodes as well as abstinence from binge eating overall in individuals undergoing CBT. When examining RCTs that used active control groups, there was no clear treatment that appeared to be superior at reducing episodes of binge eating. For BN, Svaldi *et al*. found through a search of 79 RCTs including 5,775 participants that CBT had the most effect on primary outcome variables (including remission from binge eating and compensatory behaviors, and reductions in symptom severity). These meta-analyses support the use of CBT to treat BN and BED while indicating a need for further research into CBT for BED to interpret its utility above and beyond general psychotherapy.

The transdiagnostic approach to conceptualizing treatment for eating disorders has continued to expand with increased recognition for previously under-identified diagnoses. ARFID is one such example. A newly defined diagnosis in the Diagnostic and Statistical Manual of Mental Disorders, Fifth Edition (DSM-5) ^8^, ARFID expanded upon DSM-IV’s “Feeding and Eating Disorder of Early Childhood” to better describe the condition of avoidant or restrictive eating not associated with body weight and shape concerns that may present across the lifespan. The new diagnostic category has led clinicians to begin data collection regarding assessment and treatment of individuals who meet criteria for ARFID. Thomas and Eddy ^9^ adapted the CBT model to ARFID and developed a treatment specifically tailored for the disorder, recently published in a formalized treatment manual. This model is currently being studied in treatment settings and shows initial case study support ^10^. By employing the transdiagnostic CBT model in designing treatments, researchers have been able to address a wider variety of eating disorder needs, including those of more recently defined disorders, such as ARFID.

For adolescents with AN, FBT remains a first-choice therapy owing to its demonstrated efficacy in the treatment of young patients ^11^. Earlier evidence reported that early response to treatment, specifically initial weight gain, likely predicts end-of-treatment (EOT) remission and treatment outcomes ^12, 13^. The growing empirical literature continues to suggest that early intervention and weight gain can vastly improve outcomes for adolescents with AN. As a result of FBT’s demonstrated effectiveness, research is now building upon it and extending its reach to various other clinical settings and populations, with positive support.

Recently, Le Grange and colleagues demonstrated that among 121 adolescents with AN, early weight gain predicted improved outcome at EOT in an RCT comparing FBT and individual adolescent-focused therapy (AFT) ^14^. The authors found that weight gain of 5.8 pounds by session three of FBT and 7.1 pounds by session four of AFT predicted remission status at EOT. Early weight gain did not predict remission at 6- and 12-month post-treatment follow-up for either treatment, but a survival analysis of weight gain showed FBT to be superior to AFT at 12 months post-treatment. Initial weight gain continues to be a predictive marker of treatment trajectory for adolescents with AN, with FBT maintaining strong empirical support for use in outpatient settings.

Although FBT was originally developed to be delivered in an outpatient setting, recent evidence has supported using the model within treatment programs offered at higher levels of care ^15– 20^. Matthews *et al*. reported that 49 hospitalized participants receiving an FBT-guided intervention gained significantly more weight at 3- and 6-months post-discharge ( *P* <0.05 and *P* <0.01, respectively) than a group of 44 patients examined retrospectively who received treatment as usual (TAU) ^21^. Patients who received FBT while hospitalized were also less likely to be re-hospitalized in the 6 months following discharge. Though this was only a pilot study and slightly limited by the retrospective TAU design, evidence for FBT is indicative of clinical utility at all levels of treatment, not just in the outpatient setting.

While the evidence base for FBT in adolescents with AN grows, many patients are still unable to access this intervention for reasons that may include regional differences in numbers of FBT-trained clinicians. In an effort to address this accessibility gap, Le Grange *et al*. ^22^ conducted a small study examining the feasibility of delivering FBT via a Telehealth platform. The authors reported that among 10 adolescents with AN or atypical AN, weight increased significantly from baseline to EOT as well as from baseline to 6-month follow-up. Measures of eating disorder severity also showed statistically significant changes during the study period.

As online-based platforms for delivering or augmenting treatment grow in popularity, research continues to test how treatment can be delivered most effectively for patients. One recent study examined an internet-based manualized version of CBT group therapy for BN delivered in a group chat setting and compared it to CBT delivered face-to-face in group therapy ^23^. The authors hypothesized that the online therapeutic group chat would not be inferior to the face-to-face group and tested this hypothesis in 149 adults with BN. Results showed that at EOT, the online group chat was inferior to face-to-face therapy at reducing the frequency of binge eating and purging but by 12-month follow-up the online group chat was mostly not inferior to the face-to-face group (it remained inferior for binge-eating frequency). Data suggest that CBT delivered in an online group chat setting may be an acceptable alternative to in-person group CBT, but it may involve a slower trajectory of change than traditional CBT. This online format for treatment may have value for those who otherwise would not have access to care, as well as those with less comorbid psychopathology, owing to longer time to improvement as well as less direct therapist interaction.

For adolescents with BN, FBT has also been shown to be an effective therapy ^24^. In a recently conducted RCT of 130 patients with BN or partial BN, participants randomized to FBT achieved significantly higher binge eating and purging abstinence rates (39% versus 20%) than participants receiving CBT adapted for adolescents (CBT-A). This statistically significant difference remained at 6-month follow-up, although abstinence rates were not significantly different by 12-month follow-up.

Preliminary evidence has also been put forth applying FBT to presentations of ARFID. Lock *et al*. ^25^ published a case report indicating that the favorable principles of FBT (e.g. parental empowerment, externalization, and reinforcement of serious medical and developmental consequences) are applicable to patients with ARFID as well. A feasibility study was also conducted by Lock *et al*. ^26^ of 28 patients with ARFID who were enrolled in either FBT or TAU to examine the appropriateness of conducting an RCT as well as differences in effect size between groups on clinical outcomes. In terms of weight gain, researchers found that both underweight and normal-weight patients gained more weight in FBT-ARFID (Cohen’s d = –0.90 and –0.69, respectively) than in TAU. ARFID severity scores, according to the Pica, ARFID, and Rumination Disorder Interview (PARDI), showed a greater reduction in severity (d = 0.83). Results also showed strong differences for change in parental self-efficacy favoring FBT-ARFID (d = –1.49). These results, taken with other supporting evidence for FBT in a variety of settings and across diagnoses, reinforce the need for continued study as well as more large-scale RCTs to promote the use of FBT in clinical settings.

Third-wave behavioral therapies such as dialectical behavior therapy (DBT) and acceptance and commitment therapy (ACT) have also been adapted to treat eating disorders and in recent years have been accumulating data to support their use in treatment. Although originally developed for the treatment of borderline personality disorder (BPD) ^27^, DBT for eating disorders relies on both theory and evidence of emotion dysregulation in eating disorder patients to suggest its utility ^28^. Recent data support the use of DBT in the treatment of BN and BED, but, as yet, there have been no RCTs of DBT for AN ^29^.

ACT has not yet gathered enough empirical support to be regarded as an evidence-based treatment for eating disorders ^30^. The ACT formulation appears to be well suited to addressing some of the underlying maladaptive cognitions and behaviors that are associated with eating disorders ^31^. Yet, despite earlier case studies with preliminary support for the treatment ^32^, more recent evidence has not shown ACT to perform above and beyond TAU ^33^. In 2017, Juarascio *et al.* ^34^ published results of a pilot study examining the effects of an ACT adaptation for BED. The ACT formulation, which also incorporated elements of both CBT and DBT, showed favorable results, although reported rates of binge-eating abstinence were similar to those observed with CBT alone. Before existing as a stand-alone treatment, ACT needs further development and evidence for use in eating disorder populations.

Another meta-analysis took interest in examining the effects of psychotherapy on self-esteem in BN and BED ^35^. Individuals with eating disorders are often conceptualized as having deficits in self-esteem, which has been regarded as a potential maintaining mechanism of the disorders themselves ^36^. The authors wanted to understand how self-esteem might be improved following treatment and which, if any, current psychotherapies produce significant improvements in self-esteem. Initially, the authors found modest improvements in self-esteem for BN and BED across 34 RCTs ( *g* = 0.44 and 0.20, respectively), although the authors reported that there was some evidence that the effect sizes were overestimated because of publication bias. In contrast to some previous results indicating the utility of CBT, this meta-analysis found no evidence to support CBT over non-CBT interventions for improving self-esteem. The study was limited by its lack of statistical power, so further studies examining the effect of psychotherapy on self-esteem are still indicated.

The empirical findings supporting the use of psychotherapies in the treatment of eating disorders have been reflected in the National Institute for Health and Care Excellence (NICE) guidelines, published in 2017 ^37^. They recommend group or individual CBT for AN, BN, and BED as well as family therapy (when indicated) for adolescents with AN and BN. As the evidence base accumulates for efficacious interventions, clinical recommendations will refine to reflect empirically indicated practices.

## Medications

Since the identification of distinct categories of eating disorders, pharmacologic interventions have been considered, and RCTs have been conducted to examine the potential benefit of various classes of medication on the core symptoms of eating disorders. The evidence base for antidepressant medications in the treatment of BN and BED has been robust, with outcomes including significant decrease of binge eating and purging behaviors. While fluoxetine is the only medication to have undergone the US FDA approval process that concluded with a specific indication for treatment of BN, many other antidepressants, from both the SSRI and TCA medication classes, have demonstrated statistically and clinically significant superiority compared to placebo at decreasing the frequency of binge eating and purging behaviors ^38– 45^. Notably, in a study of 387 women with BN, 60 mg fluoxetine demonstrated improved outcome compared with placebo while 20 mg did not ^46^, suggesting that a higher dose than that commonly utilized for depression is needed in the treatment of BN symptoms.

While antidepressant medications, including fluoxetine, are moderately helpful to individuals with BED in decreasing the frequency of binge eating, they are not associated with weight change effects, leaving many overweight and obese patients with BED interested in adjunctive strategies for their symptoms. BED has been described and studied since its inclusion in DSM-IV as a diagnosis in need of further research but was only recently identified as a formal clinical diagnosis with the publication of DSM-5, in 2013 ^8, 47^, and, with this renewed introduction, became the subject of additional investigations to identify helpful strategies for its management. Among these lines of investigation, lisdexamfetamine, a stimulant medication with US FDA indications for the treatment of attention deficit hyperactivity disorder (ADHD), was examined for possible utility in BED. In a randomized double-blind, parallel-group, forced dose titration, placebo-controlled clinical trial at 30 sites, 514 individuals with BED received lisdexamfetamine dimesylate at dosages of 30, 50, and 70 mg or placebo over an 11-week study period ^48^. At week 11, statistically significant differences in binge eating days/week decreased with the 50 mg/day and 70 mg/day groups but not the 30 mg/day compared with placebo. Adverse effects were consistent with the known safety profile of the medication. Additionally, the mean (SD) change in body weight was –0.1 (3.09) for the placebo group, –3.1 (3.64) kg for the 30 mg/day treatment group, –4.9 (4.43) kg for the 50 mg/day treatment group, –4.9 (3.93) kg for the 70 mg/day treatment group, and –4.3 (4.09) kg for the combined treatment group (0.001 for each dose versus placebo group comparison in post hoc analysis). Additional studies have confirmed the efficacy of 50 and 70 mg doses in binge eating days and associated symptoms, leading to approval by the FDA for the treatment of BED.

In contrast to BN and BED, AN has posed more of a clinical challenge in medication trials. In efforts to treat symptoms frequently associated with AN, including low mood, anxiety, and obsessionality, and the eating behavioral symptoms that overlap with those seen in BN, antidepressant and other medications have been examined in multiple RCTs without success.

With the development and use of atypical antipsychotic medications that help with psychiatric symptoms including severe anxiety, and are associated with weight gain in other clinical populations including those with psychotic disorders, there has been interest in the eating disorders field about whether these agents may be helpful for individuals with AN. After several small pilot studies suggesting possible benefit of olanzapine in AN ^49– 53^, Attia *et al*. examined the effects of 16 weeks of olanzapine versus placebo among 152 outpatients with AN across five clinical sites in North America ^54^. The authors found that at 16 weeks following randomization, olanzapine was associated with a statistically significant increase in BMI over time compared with that seen in the olanzapine group (0.256 [SD = 0.051] compared with 0.095 [SD = 0.052] per month). The group did not find psychological symptom differences between groups using Yale-Brown Obsessive Compulsive Scale (YBOCS), Center for Epidemiologic Studies Depression Scale (CES-D), Zung Anxiety Inventory, or Eating Disorders Examination (EDE) but did find statistically significant symptom changes associated with olanzapine on a somatic symptom inventory, with trouble concentrating, difficulty sitting still, trouble falling asleep, and trouble staying asleep being rated as more problematic in the placebo group than the group receiving olanzapine.

## Neuromodulation and neuroimaging

Advances in the understanding of the neurocircuitry involved in eating disorders has led to an increased interest in using neuromodulation as a possible treatment, especially for those with severe illness, including those who may be resistant to other established treatments ^55^. Recent research has begun to identify neural circuits that are thought to be altered among individuals with eating disorders ^56, 57^. Specifically, neurocognitive and neuroimaging data have implicated circuits involved in reward learning ^58– 61^, decision making ^62, 63^, stress, affect and negative valence ^64, 65^, appetite regulation ^66, 67^, and self-regulatory control ^58, 67^. Understanding these circuits and their locations in the brain has been critical for identifying treatment targets that may be used for treatment development with neuromodulation technologies.

Current methods for neuromodulation range from relatively non-invasive modalities like repetitive transcranial magnetic stimulation (rTMS) to more intensive/invasive types such as deep brain stimulation (DBS).

rTMS has been examined as both a probe to test neurobiological hypotheses and a possible therapeutic intervention. One 2016 case series of five adult women with AN measured changes in visual analogue scales (VAS), EDE-Q scores, and BMI before and after 20 sessions of rTMS to the left dorsolateral prefrontal cortex (l-DLPFC) ^68^. Results showed overall lower VAS scores across levels of stress, anxiety, urges to restrict, and feelings of fullness post-TMS. There was no notable change in BMI from pre- to post-treatment for the patients, but eating disorder symptomatology (as measured by the EDE-Q) improved and persisted up to the 12-month follow-up. This case study was followed by a larger RCT of 49 patients with AN who were randomized to receive either real or sham rTMS to the l-DLPFC ^69^. Patients filled out baseline measures of eating disorder and general psychopathology and then were asked to complete a food challenge task (FCT), which involved watching a video of people eating palatable foods with those same foods in the room, before filling out VAS and receiving the real or sham rTMS treatment. After the rTMS, patients repeated the FCT and VAS, along with measures of temporal discounting and saliva samples. Primary outcomes were measured by the VAS and described as “core AN symptoms”: urges to restrict, levels of feeling full, and levels of feeling fat. Results indicated a trend for group differences in the effects of rTMS on core AN symptoms ( *P* = 0.056). While notably the effects of TMS are transient in comparison to other therapeutic interventions, initial evidence provides modest support that TMS may allow for symptom reduction in patients with AN.

The more intensive intervention, DBS, has been used in an open series of 16 patients with treatment-refractory AN and promising results have been reported ^70^. DBS treatment targeting the subcallosal cingulate cortex was associated with improved anxiety, depression, emotion regulation, and BMI at 12 months post-surgery. Other case reports of DBS for AN have described similarly promising results, but controlled studies of this intervention are needed to determine the impact of this strategy ^71– 73^.

Many of these data come from preliminary studies in the field, with mixed results suggesting that the clinical utility of these interventions has yet to be fully understood. With the advancement of neuroimaging data enhancing our knowledge of the neurobiological underpinnings of psychiatric illnesses, neuromodulatory targets will continue to be explored.

## Conclusion

There have been considerable advances in therapies for eating disorders within the past few years alone. Relying on the recent transdiagnostic CBT-E model of eating disorders ^4^, CBTs continue to show support for patients with BN and BED ^5^. Empirical data indicate that the model can be formulated to fit diagnoses, with promising results ^10^. FBT for adolescents has been studied across a variety of contexts ^15– 22^ and multiple diagnoses ^14, 24, 26^ with favorable results, underscoring its clinical utility in young patients. Medication trials have led to the FDA approval of lisdexamfetamine for the treatment of BED ^48^, and there is promise for olanzapine as an adjunctive treatment for weight gain in AN ^49– 54^. Neuroimaging studies have laid the groundwork for understanding underlying brain mechanisms that may contribute to the development and perpetuation of eating disorders, with neuromodulatory therapies currently being piloted as possible targeted interventions ^68– 70^. Further research and development are still needed to refine the clinical utility of many treatments, but favorable outcomes point in the direction of progress toward empirically defined, effective eating disorder treatments.
